# Editorial: Exploiting the Immune System to Treat Multiple Myeloma: From Transplantation to Novel Treatment Approaches

**DOI:** 10.3389/fonc.2020.607571

**Published:** 2020-10-06

**Authors:** Niels W. C. J. van de Donk, Efstathios Kastritis, Francesca Gay

**Affiliations:** ^1^Amsterdam UMC, Vrije Universiteit Amsterdam, Department of Hematology, Cancer Center Amsterdam, Amsterdam, Netherlands; ^2^Plasma Cell Dyscrasia Unit, Department of Clinical Therapeutics, National and Kapodistrian University of Athens, Athens, Greece; ^3^Division of Hematology, Citta della Salute e della Scienza, University of Torino, Torino, Italy

**Keywords:** myeloma, immunotherapy, immunoconjugate, CAR (chimeric antigen receptor) T cells, antibody

During the last two decades, the survival of multiple myeloma (MM) patients has markedly improved due to the introduction of proteasome inhibitors (PIs; bortezomib, ixazomib, and carfilzomib) and immunomodulatory drugs (IMiDs; thalidomide, lenalidomide, and pomalidomide). In younger patients high-dose therapy and autologous stem cell transplantation have also contributed to prolonged disease control. More recently several new immunotherapeutic agents are transforming MM treatment. Naked monoclonal antibodies (defined as antibodies which have no drug or radionuclide attached to their Fc tail) were the first immunotherapeutic agents evaluated in MM, and several of these antibodies are now approved for the treatment of MM. First, the SLAMF7-targeting antibody, elotuzumab, can be effectively and safely combined with lenalidomide-dexamethasone (1) and pomalidomide-dexamethasone in relapsed/refractory MM (2). However, until now addition of elotuzumab to standards-of care in newly diagnosed MM (NDMM) patients failed to improve response rate, progression-free survival (PFS) or overall survival (OS) (3, 4). In contrast, addition of the first-in-class CD38 targeting antibody daratumumab to backbone regimens has shown to improve response rates, PFS, and in some studies, also OS in both the newly diagnosed (5–8) and relapsed/refractory settings (9–11). Other CD38 antibodies such as isatuximab and TAK-079 are also effective in RRMM as single agent (12, 13). Moreover, recent phase 3 trials showed that addition of isatuximab to carfilzomib-dexamethasone or pomalidomide-dexamethasone improves response rate and PFS (14, 15).

In this issue, the paper entitled “The Role of Immunotherapy in Non-transplant Eligible Multiple Myeloma” by Bobin et al. describes how these SLAMF7 and CD38-targeting antibodies improve the outcome not only in young, but also in elderly MM patients. This is an important observation, since the improvement in outcome in elderly, non-transplant eligible patients, is less pronounced than in younger patients, most likely due to their increased susceptibility to treatment toxicity, which frequently results in treatment discontinuation (16). Indeed, elotuzumab and the CD38 antibodies proved to be well tolerated in both younger and elderly patients, also when combined with other anti-MM agents (17).

Patients, who develop disease that is resistant to PIs, IMiDs, and CD38 antibodies (triple-class refractory), have a very poor outcome, especially patients refractory to 2 PIs, 2 IMIDs, and a CD38 antibody (penta-refractory patients; median OS: 5.6 months) (18). The FDA and EMA recently approved belantamab mafodotin for the treatment of patients with relapsed or refractory MM who have received at least four prior therapies including an IMiD, a PI, and a CD38 antibody. Belantamab mafodotin is an antibody-drug conjugate that consists of a humanized B cell maturation antigen (BCMA)-specific IgG1 mAb fused to monomethyl auristatin F (MMAF) by a non-cleavable linker. Bruins et al. describe in their paper entitled “Targeted Therapy With Immunoconjugates for Multiple Myeloma” that belantamab mafodotin kills MM cells not only *via* the toxic effects of the microtubule disrupting agent MMAF, but also by immune-mediated mechanisms of action such as antibody-dependent cellular cytotoxicity (ADCC) and antibody-dependent cellular phagocytosis (ADCP). These pleiotropic mechanisms of action result in an approximately 30% response rate in triple-class refractory patients (19). Moreover, antibodies can be utilized as carriers of other effector moieties such as toxins, cytokines, or radionuclides. Bruins et al. also discuss the mechanisms of action, safety, and efficacy of several other promising immunoconjugates that are under investigation in preclinical and/or clinical MM studies.

In addition, chimeric antigen receptor (CAR) T-cells hold great promise for extensively pretreated MM patients. High response rates are obtained with BCMA-specific CAR T-cells (20, 21). However, studies with longer follow-up failed to show a plateau in the survival curves, indicating that CAR T-cell therapy needs further improvement. Van der Schans et al. describe in the manuscript entitled “Dual targeting to overcome current challenges in multiple myeloma CAR T-cell treatment” how CAR T-cell therapy can be improved in MM. The authors review other targets for CAR T-cell therapy and discuss how dual CAR targeting may lead to improved clinical outcomes by tackling target antigen loss or downregulation and by allowing the use of MM-associated, but not specific, target antigens.

The incorporation of new immunotherapeutic drugs in the treatment of MM has resulted in an increased rate of high-quality responses. However, several studies have shown that not all patients with a complete response, whereby light microscopy is used to define the percentage of tumor cells in the bone marrow, experience a prolonged survival (22). This indicates that more sensitive techniques are needed to detect the presence of tumor cells in the bone marrow. At this moment, minimal residual disease (MRD) can be detected by either multi-parameter flow cytometry or by next-generation sequencing, which reliably achieves 10^−5^ to 10^−6^ sensitivity for MM cell detection. Kostopoulos et al. discuss in their paper entitled “Minimal Residual Disease in Multiple Myeloma: Current Landscape and Future Applications With Immunotherapeutic Approaches” that MRD can be used as a prognostic factor, and they review how several trials are currently using MRD assessment to tailor treatment (*e.g.* guidance for type and duration of maintenance treatment).

Finally, the manuscript “Deregulation of Adaptive T Cell Immunity in Multiple Myeloma: Insights Into Mechanisms and Therapeutic Opportunities” by Leblay et al. describes how MM cells can escape immune-mediated attack in the immune-suppressive bone marrow microenvironment. In their review, novel insights are provided into the mechanisms that promote tumor escape, cause inadequate T-cell stimulation and impaired cytotoxicity in MM. Furthermore, the review highlights how adaptive T-cell immune responses can be restored in MM. A better understanding of these immune evasion strategies has resulted in the identification of novel targets for immunotherapy in MM. We expect that these insights will eventually lead to new immunotherapeutic strategies and further improvement in the survival of MM patients.

Overall, the different contributions show that immunotherapy has changed MM treatment and that in the nearby future the introduction of new immunotherapeutic approaches such as CAR T-cells, immunoconjugates, and bispecific antibodies, together with the use of more sensitive techniques to evaluate disease-response, will result in further improvement in the outcome of MM patients.

## Author Contributions

All authors contributed to the article and approved the submitted version.

## Conflict of Interest

ND has received research support from Janssen Pharmaceuticals, AMGEN, Celgene, Novartis, and BMS and serves in advisory boards for Janssen Pharmaceuticals, AMGEN, Celgene, BMS, Takeda, Roche, Novartis, Bayer, and Servier. EK has received honoraria/personal fees from Amgen, Genesis Pharma, Janssen, Takeda and Prothena and research grants from Amgen and Janssen. FG has received honoraria from Janssen Pharmaceuticals, AMGEN, Celgene, BMS, Takeda, Sanofi and serves in advisory boards for Janssen Pharmaceuticals, AMGEN, Celgene, BMS, Takeda, Roche, Abbvie, Sanofi, Oncopeptides and Adaptive.
